# The impact of algorithm-driven exposure to disease-related short videos on rehabilitation outcomes in lumbar disc herniation patients: content heterogeneity and psychological mediating mechanisms

**DOI:** 10.3389/fdgth.2026.1774844

**Published:** 2026-03-02

**Authors:** Yiping Tong, Yang Li, Chenxi Liu, Xiang Chen, Linbo Xing, Zhiyuan Cao, Yanlei Wang

**Affiliations:** 1 Luoyang Orthopedic Hospital of Henan Province (Henan Provincial Orthopedic Hospital), Luoyang, China; 2 School of Nursing, Fujian University of Traditional Chinese Medicine, Fuzhou, China; 3 Henan University Henan Provincial Center for Applied Mathematics, Zhengzhou, China

**Keywords:** health anxiety, lumbar discmHerniation (LDH), lumbar functional rehabilitation, recommendation algorithm, short videos

## Abstract

**Background:**

Short videos have become a primary channel for Lumbar Disc Herniation (LDH) patients to obtain disease knowledge and rehabilitation guidance. Algorithm-driven personalized recommendations may expose patients to heterogeneous LDH-related content, affecting their health anxiety and rehabilitation trajectories.

**Objective:**

This study explored the impacts of LDH-related short video exposure duration and content types on health anxiety and lumbar functional rehabilitation in LDH patients, and verified the mediating role of health anxiety.

**Methods:**

A 6-month prospective cohort study enrolled 213 LDH outpatients from Luoyang Orthopedic-Traumatological Hospital (Jan–Apr 2025). Demographic, clinical and short video usage data were collected. Health anxiety (MCQ-HA) and lumbar function (JOA) were assessed at baseline and follow-up. Pearson correlation, multiple linear regression, subgroup analysis and Bootstrap mediation analysis (5,000 resamplings) were used.

**Results:**

At 6-month follow-up, the mean JOA score decreased from 23.00 ± 1.59 at baseline to 21.96 ± 3.03, and the mean MCQ-HA score increased from 20.77 ± 4.57–21.86 ± 6.14. Pearson correlation analysis showed that daily viewing duration and exposure frequency to awareness-motivation content were significantly negatively correlated with *Δ*JOA (*r* = –0.36, *r* = –0.33; both *P* < 0.001) and positively correlated with *Δ*MCQ-HA (*r* = 0.31, *r* = 0.34; both *P* < 0.001). Multiple linear regression indicated that *Δ*JOA in the >60 min daily viewing group was significantly lower than that in the <30 min group; exposure frequency to awareness-motivation content was independently negatively associated with *Δ*JOA and positively associated with *Δ*MCQ-HA (both *P* < 0.001), with no significant associations found for other content categories (all *P* > 0.05). Subgroup analysis based on clinical efficacy criteria revealed significant differences in recovery outcomes across viewing duration groups (*χ*^2^ = 18.75, *P* = 0.004). Bootstrap mediation analysis confirmed that *Δ*MCQ-HA mediated 16.13% of the total effect of daily viewing duration on *Δ*JOA and 20.80% of the total effect of awareness-motivation content exposure frequency on *Δ*JOA.

**Conclusion:**

Prolonged short video exposure and frequent awareness-motivation content viewing were associated with poorer rehabilitation and higher health anxiety, with health anxiety partially mediating these relationships, providing empirical evidence for digital health guidance.

## 1 Introduction

Empowered by digital technologies, short videos have profoundly reshaped the overall landscape of information dissemination and consumption. According to the 56th Statistical Report released by the China Internet Network Information Center (CNNIC), as of June 2025, the user base of short videos in China has reached 1.068 billion, with an average daily viewing duration of approximately 151 min. This data indicates that short videos have evolved from a mere form of entertainment to a basic information service carrier covering the entire population (1). Its core competitiveness stems from “personalized recommendation” algorithms based on artificial intelligence (AI) technology: by deeply analyzing users’ browsing trajectories and interaction preferences, these algorithms construct tailored content streams with a “one-size-fits-none” approach. This not only significantly reduces users’ information search costs but also creates a highly immersive user experience (2, 3). As a globally leading social media platform, TikTok has built its content ecosystem by leveraging such recommendation algorithms, encouraging user participation in short video creation and sharing. Statistics from 2023 indicate that its global cumulative active users have reached 2 billion, with Monthly Active Users (MAU) exceeding 900 million (4). For patients with chronic diseases who require long-term disease self-management, short video platforms, by virtue of their convenience and personalized content advantages, have naturally become an important channel for them to access disease knowledge, rehabilitation guidance, and emotional support (5).

Lumbar disc herniation (LDH) is the most common disorder in spinal surgery, characterized by high prevalence, a prolonged disease course, and a high recurrence rate. The efficacy of its treatment and rehabilitation is highly dependent on patients’ long-term self-management behaviors and accurate disease perception (6, 7).Traditional health information access channels, such as doctor-patient communication, printed materials, or static webpages, are being supplemented or even partially replaced by dynamic, intuitive, and highly engaging short video content. Driven by the need to alleviate symptoms and seek rehabilitation methods, patients are actively or passively immersed in the information streams constructed by algorithms (8). This health information environment, driven by data and code, harbors non-negligible risks: the platform's recommendation logic is based on “user interaction data” and “traffic preferences” rather than “hierarchical medical evidence,” which may lead patients to be systematically exposed to an information environment with mixed quality—where scientific rehabilitation guidance coexists with unproven miracle cures and exaggerated commercial content. Furthermore, prolonged immersion in lumbar disease-related videos may exacerbate patients’ anxiety or disease burden. Bright et al. (9) found that over-reliance on short video recommendation algorithms may cause users to overlook their own intuitive feelings and judgments, or information overload may lead to decision fatigue, which to a certain extent increases users’ stress and anxiety. When this general mechanism acts on patients seeking clear rehabilitation plans, its negative effects may be amplified: patients may fall into cognitive confusion and psychological distress amid contradictory, intimidating, or false information.

Despite the increasingly prominent role of short videos in health communication, relevant academic research remains underexplored. First, most studies have remained confined to cross-sectional analyses of platform content quality or user attitude surveys (8, 10), with a critical lack of longitudinal causal evidence regarding the impact of immersion duration and content exposure on objective clinical outcomes. Second, existing research mostly treats social media use as a monolithic construct, failing to distinguish between the differential impacts that specific algorithm-recommended content types and immersive usage behaviors may independently exert. Finally, in chronic disease management contexts such as LDH, there is a paucity of in-depth empirical examination into whether and how health anxiety functions as a key mediating variable linking digital information behaviors to physical rehabilitation outcomes. Thus, this study designed and conducted a 6-month prospective cohort study. The primary objective is to systematically analyze how immersion duration in relevant short videos and algorithm-recommended content types independently and synergistically influence the symptoms of LDH patients by affecting health anxiety.

## 2 Materials and methods

### 2.1 Study design and informed consent

This was a 6-month prospective cohort study designed to examine the associations between the content and duration of recommendations from short video platforms and the severity of symptoms in patients with LDH. Baseline data were collected from January to April 2025, with a telephone follow-up conducted 6 months later. A total of 213 patients diagnosed with LDH were recruited from the outpatient department of Luoyang Orthopedic-Traumatological Hospital of Henan Province (Henan Orthopedic Hospital). Prior to enrollment, all participants were provided with detailed study explanations. After fully understanding the research purpose, procedures, potential risks, and benefits, they signed written informed consent forms. The study protocol, content of the informed consent form, and data collection procedures were reviewed and approved by the Human Research Ethics Committee of Luoyang Orthopedic-Traumatological Hospital of Henan Province (Henan Orthopedic Hospital) (Ethics Approval No.: 2023XJS0005-01). The entire study was conducted in strict adherence to the Declaration of Helsinki and relevant ethical norms for medical research.

### 2.2 Study population

Recruitment was conducted from January to April 2025. All eligible outpatients diagnosed with LDH at Luoyang Orthopedic-Traumatological Hospital of Henan Province (Henan Provincial Orthopedic Hospital) were invited to participate.Inclusion Criteria: 1. Definitely diagnosed with LDH via lumbar computed tomography (CT) or magnetic resonance imaging (MRI) examination; 2. Primarily receiving conservative medical treatment; 3. Age between 18 and 65 years; 4. Possessing basic ability to use short video platforms; Exclusion Criteria: 1. Complicating with other spinal diseases such as lumbar spinal stenosis, lumbar spondylolisthesis, spinal tumor, or spinal infection; 2. Having severe cardiac, hepatic, renal, or other organ dysfunction; 3. Having cognitive impairment, mental illness, or being unable to communicate effectively.

The sample size was calculated using the formula for single-group repeated measures:

$$M=[1+(k-1)\rho ]\sigma^{2}(Z\alpha /2+Z\beta {)}^{2}/(k\delta^{2})$$

where *α* = 0.05 (Z*α*/2 = 1.96) and *β* = 0.20 (Z*β* = 0.84). Here, k represents the number of repeated measurements (k = 2 in this study). Based on a study by Wang et al (11) investigating the impact of short video platform recommendation algorithms on users’ perceived stress and health management behaviors, ρ was set to 0.51, σ to 2.00, and δ to 0.37, resulting in a calculated sample size of 173. Accounting for a 20% attrition rate, a minimum of 208 participants was required.

### 2.3 Primary independent variables

#### 2.3.1 Time to monitor related conditions

This variable was optimized based on previous research methods and tailored to the core needs of the present study. Researchers administered the following survey question to participants: “Over the past month, approximately how long did you spend using short video apps to watch content related to LDH and other spinal conditions on a typical weekday/weekend day?” Participants were required to report their average daily viewing duration on weekdays and weekends separately, in hours and minutes. To balance the differences in viewing patterns between weekdays and weekends, the weekly average daily viewing duration (ST) was calculated using the formula: (Average daily weekday duration × 5 + Average daily weekend duration × 2) ÷ 7.

#### 2.3.2 Categories of recommended content

**Science Education**: Content grounded in medical evidence that objectively explains the aetiology, pathology, standard treatment, and rehabilitation of LDH. **Experience Sharing**: Content in which patients or individuals in recovery share personal illness narratives, rehabilitation journeys, and daily life adaptation strategies. **Awareness-Motivation**: Content designed primarily to elicit vigilance by emphasizing negative outcomes associated with LDH, such as severe complications from delayed treatment, risks of suboptimal rehabilitation, and fears related to surgical intervention. **Marketing-Referral**: Commercial content aimed at promoting pharmaceuticals, medical devices, physiotherapy services, or other health-related products and services.

Data Issue Collection: Over the past 7 days, when browsing the “For You” or “Discovery” pages of short video platforms (e.g., TikTok, Quick Hands), how frequently did the platform's algorithm automatically recommend LDH-related content including science popularization and education content, experience sharing content, warning and mobilization-related content, and marketing and traffic diversion content? (1. Almost every day; 2. Several times a week; 3. Occasionally; 4. Almost never)

To ensure the reliability of content categorization, a pilot study was conducted prior to the main analysis. Two trained researchers independently coded 50 randomly selected LDH-related short videos. Inter-rater agreement was assessed using Cohen's kappa statistic, yielding a value of 0.83 (*P* < 0.001), which indicates excellent reliability of the classification framework.

### 2.4 Primary dependent variables

Change in Health Anxiety (*Δ*MCQ-HA): This variable was assessed using the Metacognitions Questionnaire for Health Anxiety (MCQ-HA), a standardized 14-item assessment tool (12). The scale focuses on individuals’ metacognitive beliefs and attitudes toward health anxiety, including pathogenic worry preoccupation, biased thinking beliefs, and uncontrollability of thoughts beliefs, with a total score ranging from 0 to 56—higher scores indicate higher metacognitive levels related to health anxiety (13). Assessments were conducted at baseline (T0) and 6 months later (T1), and *Δ*MCQ-HA was calculated as T1MCQ-HA minus T0MCQ-HA.

Change in Lumbar Function (*Δ*JOA): Lumbar function was evaluated using the Japanese Orthopaedic Association (JOA) Lumbar Functional Score (14), which assesses patients with low back pain across three dimensions: subjective symptoms, clinical signs, and limitations in daily activities. The total JOA score ranges from 0 to 29, with scores categorized as follows: <10 = poor; 10–15 = moderate; 16–24 = good; and 25–29 = excellent. *Δ*JOA was computed as T1JOA minus T0JOA. The improvement rate was calculated using the formula: [*Δ*JOA ÷ (full score of 29−T0JOA)] × 100%. This rate was further aligned with conventional clinical efficacy criteria: 100% improvement = cured; >60% improvement = marked improvement; 25%–60% improvement = improvement; and <25% improvement = no improvement.

### 2.5 Covariates

Covariates included demographic characteristics (age, gender, educational level, marital status, monthly income, BMI); disease-related characteristics (disease duration, herniated segment, symptom types: low back pain/lower limb pain/numbness, comorbidities); lifestyle habits (smoking history, alcohol consumption history); and short video usage-related factors (short video platforms used, types of health accounts followed).

### 2.6 Statistical methods

All statistical analyses were conducted using R software (version 4.3.0). Continuous variables with normal distribution are presented as mean ± standard deviation (SD). Between-group comparisons for these variables were made using t-tests. Variables with non-normal distribution are presented as median (interquartile range, IQR). Between-group comparisons for non-normally distributed variables were performed using the Mann–Whitney *U* test. Categorical variables are described as frequency (percentage). Between-group comparisons for categorical variables were performed using the chi-square test. The exposure frequency of the four types of algorithm-recommended LDH-related content on short video platforms was included in the models as an ordinal categorical variable to test the robustness of the results. Pearson correlation analysis was used to explore the simple associations between short video usage behaviors, content exposure, and health outcomes in LDH patients.

To ensure the robustness of the regression results, we conducted systematic diagnostics on the final models. Specifically, variance inflation factor (VIF) values (all <5) indicated no severe multicollinearity. Scatter plots of standardized residuals against predicted values supported the assumptions of linearity and homoscedasticity. Visual inspection of Q-Q plots suggested that the residuals were approximately normally distributed. Finally, the Durbin-Watson test confirmed the independence of the residuals.

Pearson's chi-square (*χ*^2^) test was used to compare differences in the distribution of efficacy grades across the three viewing duration groups. Additionally, subgroup analysis was conducted to assess the associations between short video viewing duration and *Δ*JOA/*Δ*MCQ-HA, with stratified model fitting performed across seven prespecified subgroups: age group, gender, disease duration group, educational level, BMI group, herniated segment, and symptom type.

Finally, mediation effect analysis was conducted using the mediation package in R. “Exposure frequency to warning and mobilization-related content” and “short video viewing duration” were set as independent variables (X), *Δ*MCQ-HA as the mediating variable (M), and *Δ*JOA as the dependent variable (Y), with adjustment for the aforementioned covariates. The Bootstrap method (5,000 resamplings) was used to calculate the indirect effect; a significant mediation effect was considered present if the 95% confidence interval (95% CI) of the indirect effect did not include 0.

The significance level (α) for all statistical tests in this study was set at 0.05, and two-tailed tests were used throughout.

## 3 Results

### 3.1 Baseline characteristics

A total of 250 questionnaires were distributed, with 213 valid responses collected. Ultimately, 213 LDH patients were included in the study, comprising 106 males (49.77%) and 107 females (50.33%). The mean age of the patients was 42.23 ± 13.59 years. After 6 months of follow-up, patients’ lumbar function exhibited certain changes: the JOA score decreased from 23.00 ± 1.59 at baseline to 21.96 ± 3.03 at follow-up. Concurrently, health anxiety levels increased, with the MCQ-HA score rising from 20.77 ± 4.57 at baseline to 21.86 ± 6.14 at follow-up.

Table 1 summarizes the baseline characteristics of participants stratified into three groups based on daily viewing duration of LDH and spine-related disease videos. Results indicated that the three groups exhibited significant differences in multiple demographic and behavioral characteristics. Regarding demographic features, daily viewing duration was statistically associated with age, gender distribution, educational level, and BMI. For clinical characteristics, no statistically significant differences were observed among the three groups in terms of disease duration or distribution of affected spinal segments (all *P* > 0.05). In terms of platform usage behaviors, the primary platform used and type of accounts followed (*P* < 0.05) were unevenly distributed across the different viewing duration groups. Notably, in the group with daily viewing duration <30 min, as many as 36.84% of users did not follow any health-related accounts. Regarding health scores, no significant differences were found in MCQ-HA (*P* = 0.333) or JOA (*P* = 0.134) scores among the three groups at baseline (T0), indicating that patients in each group had comparable health anxiety levels and lumbar function status at the study initiation. However, after 6 months of follow-up, intergroup differences emerged: patients with daily viewing duration >60 min exhibited significantly higher MCQ-HA scores (*P* < 0.001) and significantly lower JOA scores (*P* < 0.001) at follow-up (T1).

**Table 1:** Demographic and clinical characteristics of 213 patients with LDH stratified by the primary exposure Variable.

Characteristics	Overall (*n* = 213)	Average time spent watching LDH and spine-related diseases (min/day)			*P*-value
		<30 (*n* = 57)	30–60 (*n* = 76)	>60 (*n* = 80)	
Age, mean (SD)	42.23 (13.59)	38.65 (12.43)	41.74 (13.73)	45.26 (13.73)	**0**.**022**
Gender, *n* (%)					**0**.**009**
Male	106 (49.77)	32 (56.14)	45 (59.21)	29 (36.25)	
Female	107 (50.33)	25 (43.86)	31 (40.79)	51 (63.75)	
Educational attainment, *n* (%)					**0**.**033**
Below junior high school level	56 (26.29)	11 (19.30)	17 (22.37)	28 (35.0)	
High School/Vocational School	74 (34.74)	18 (31.58)	25 (32.89)	31 (38.75)	
Junior college	49 (23.00)	19 (33.33)	16 (21.05)	14 (17.5)	
Bachelor's degree or higher	34 (15.96)	9 (15.79)	18 (23.68)	7 (8.75)	
BMI, mean (SD)	24.45 (3.23)	25.03 (3.14)	23.52 (3.40)	24.91 (2.93)	**0**.**007**
Monthly income, *n* (%)					**0**.**305**
<6,000	53 (24.88)	12 (21.05)	22 (28.95)	19 (23.75)	
6,000–9,000	67 (31.46)	24 (42.11)	19 (25.00)	24 (30.0)	
>9,000	93 (43.66)	21 (36.84)	35 (46.05)	37 (46.25)	
Duration of illness, mean (SD)	7.04 (3.27)	6.89 (3.14)	7.24 (3.32)	6.95 (3.34)	**0**.**764**
Affected segment, *n* (%)					**0**.**343**
L4–L5	105 (49.30)	28 (49.12)	37 (48.68)	40 (50.0)	
L5–S1	84 (39.44)	23 (40.35)	34 (44.74)	27 (33.75)	
Other	24 (11.27)	6 (10.53)	5 (6.58)	13 (16.25)	
Primary platforms, *n* (%)					**0**.**098**
TikTok	105 (29.30)	32 (56.14)	34 (44.74)	39 (48.75)	
Quick hands	69 (32.39)	20 (35.09)	23 (30.26)	26 (32.50)	
WeChat video channel	28 (13.15)	1 (1.75)	14 (18.42)	13 (16.25)	
Other	11 (51.6)	4 (7.01)	5 (6.58)	2 (2.50)	
Follow account type, *n* (%)					**0**.**014**
Patient sharing	55 (25.82)	9 (15.79)	21 (27.63)	27 (33.75)	
Rehabilitation therapist/physician	57 (26.76)	11 (19.30)	22 (28.95)	23 (28.75)	
Science popularization blogger	55 (25.82)	16 (28.07)	20 (26.32)	20 (25.0)	
Not followed	46 (21.60)	21 (36.84)	13 (17.11)	10 (12.50)	
T_0_MCQ-HA	20.77 (4.57)	20.67 (3.74)	20.24 (4.68)	21.36 (4.96)	0.134
T_1_MCQ-HA	21.86 (6.14)	20.35 (5.50)	20.64 (6.13)	24.10 (5.98)	0.333
T_0_JOA	23.00 (1.59)	23.12 (1.44)	22.72(1.74)	23.19(1.54)	<0.001
T_1_JOA	21.96(3.03)	23.18(2.85)	21.97(2.82)	21.09(3.07)	<0.001

The bold represent the P-values follows statistical testing.

### 3.2 Short video content exposure frequency across different viewing duration groups

**Figure 1:**
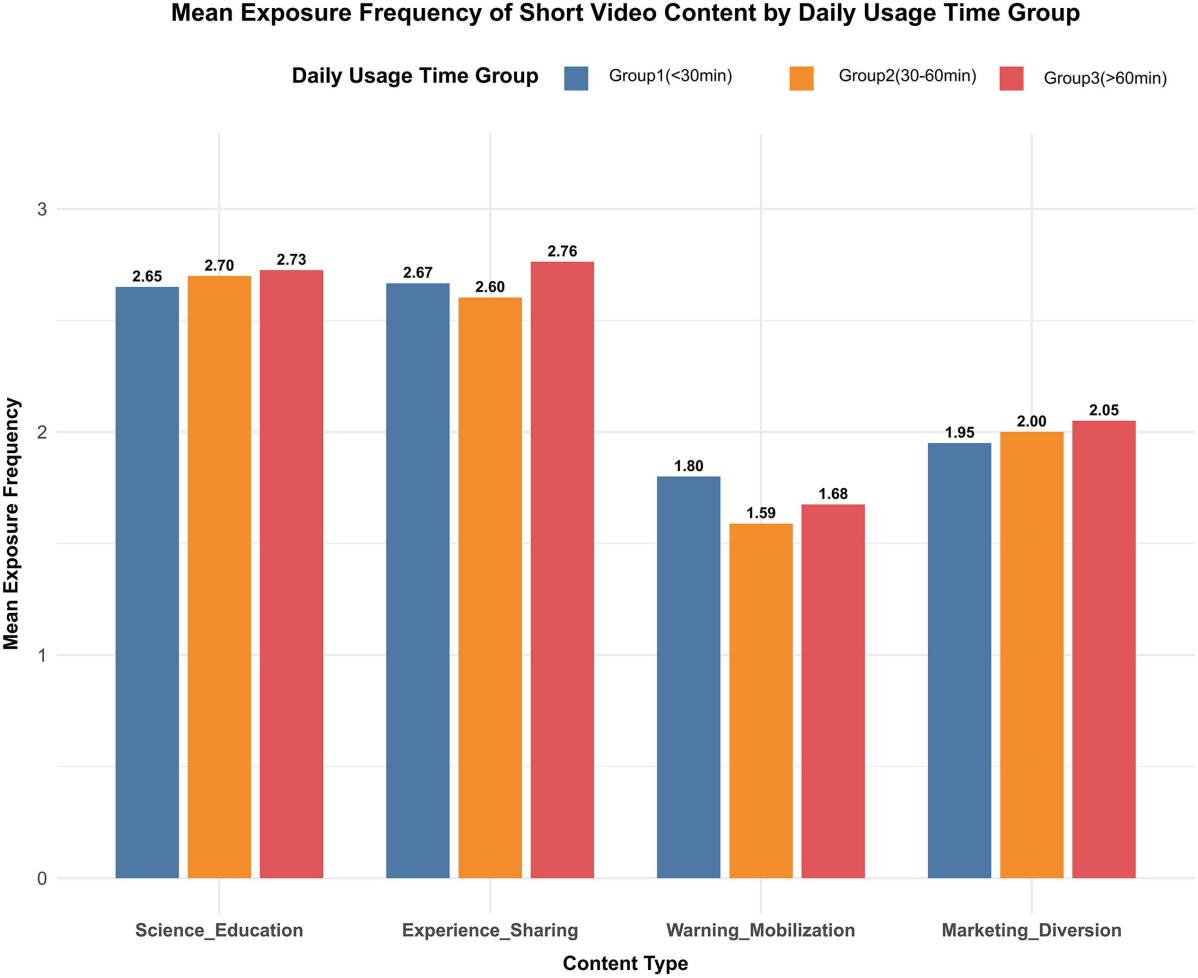
Exposure frequency of short video platform-recommended content across different viewing duration groups.

Analysis of the exposure frequency of algorithm-recommended content on short video platforms across different viewing duration groups (Figure 1) revealed that as the daily average viewing duration increased, the average exposure frequency of patients to the four types of disease-related content exhibited an upward trend. In detail, the exposure rates of popular science education content were 2.65, 2.70, and 2.73 in Group 1, Group 2, and Group 3, respectively; those of experience sharing content were 2.67, 2.60, and 2.76; those of marketing diversion content were 1.95, 2.00, and 2.05; and those of warning-mobilization content were 1.80, 1.59, and 1.68. The exposure frequency to the four categories of disease-related content exhibited an overall increasing trend with longer daily viewing time. This rising trend was more discernible for the science education, experience-sharing, and marketing-referral categories. Although the awareness-motivation category showed slight fluctuations in the medium-duration group (Group 2), no overall downward trend was observed.

### 3.3 Correlations between short video usage behaviors, content exposure, and health outcomes in LDH patients

**Figure 2:**
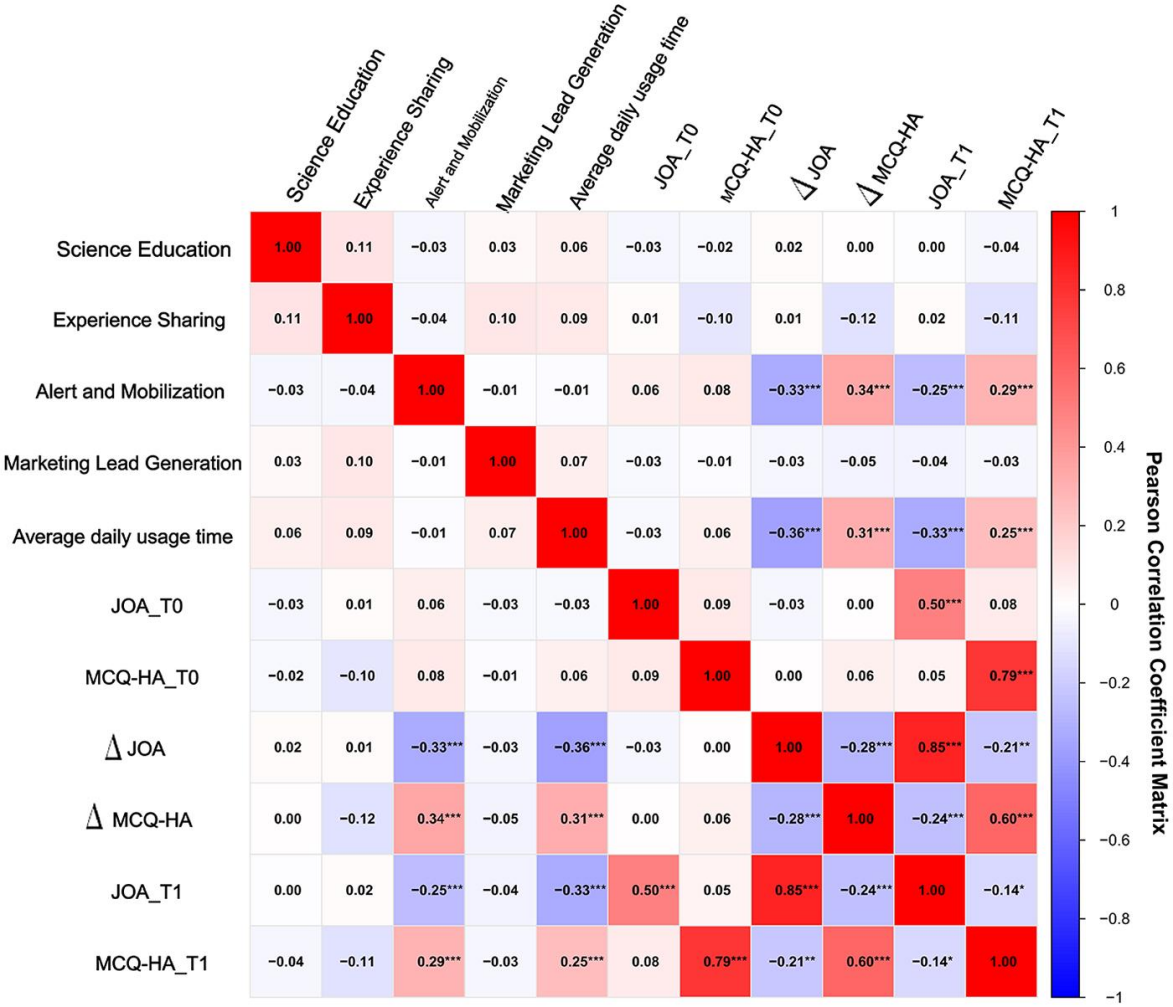
Correlation heatmap of short video usage behaviors, content exposure, and health outcomes in LDH patients. *** indicates *p* < 0.001; **indicates *p* < 0.01; * indicates *p* < 0.05. JOA_T0 is the baseline JOA score; JOA_T1 is the 6-month follow-up JOA score; *Δ*JOA is the change in JOA score; MCQ-HA_T0 is the baseline MCQ-HA score; MCQ-HA_T1 is the 6-month follow-up MCQ-HA score; *Δ*MCQ-HA is the change in metacognitive score for health anxiety.

Pearson correlation analysis was conducted to examine the associations between short video viewing behaviors, content exposure, and health outcomes among patients with LDH. The results indicated that daily viewing time of lumbar and spinal disease-related content was significantly negatively associated with both the change in JOA score (*Δ*JOA) and the T1 JOA score (*r* = −0.36, *p* < 0.001; *r* = −0.33, *p* < 0.001). Conversely, it showed significant positive associations with the change in health anxiety (*Δ*MCQ-HA) and the T1 MCQ-HA score (*r* = 0.31, *p* < 0.001; *r* = 0.25, *p* < 0.001). Furthermore, a significant negative correlation was observed between *Δ*JOA and *Δ*MCQ-HA (*r* = −0.28, *p* < 0.001).Regarding content categories, exposure frequency to awareness-motivation content showed significant negative associations with both *Δ*JOA and T1 JOA scores (*r* = −0.33, *p* < 0.001; *r* = −0.25, *p* < 0.001), and significant positive associations with both *Δ*MCQ-HA and T1 MCQ-HA scores (*r* = 0.34, *p* < 0.001; *r* = 0.29, *p* < 0.001). For details, see Figure 2.

### 3.4 Effects of short video usage behaviors on *Δ*JOA and *Δ*MCQ-HA scores in LDH patients

**Figure 3:**
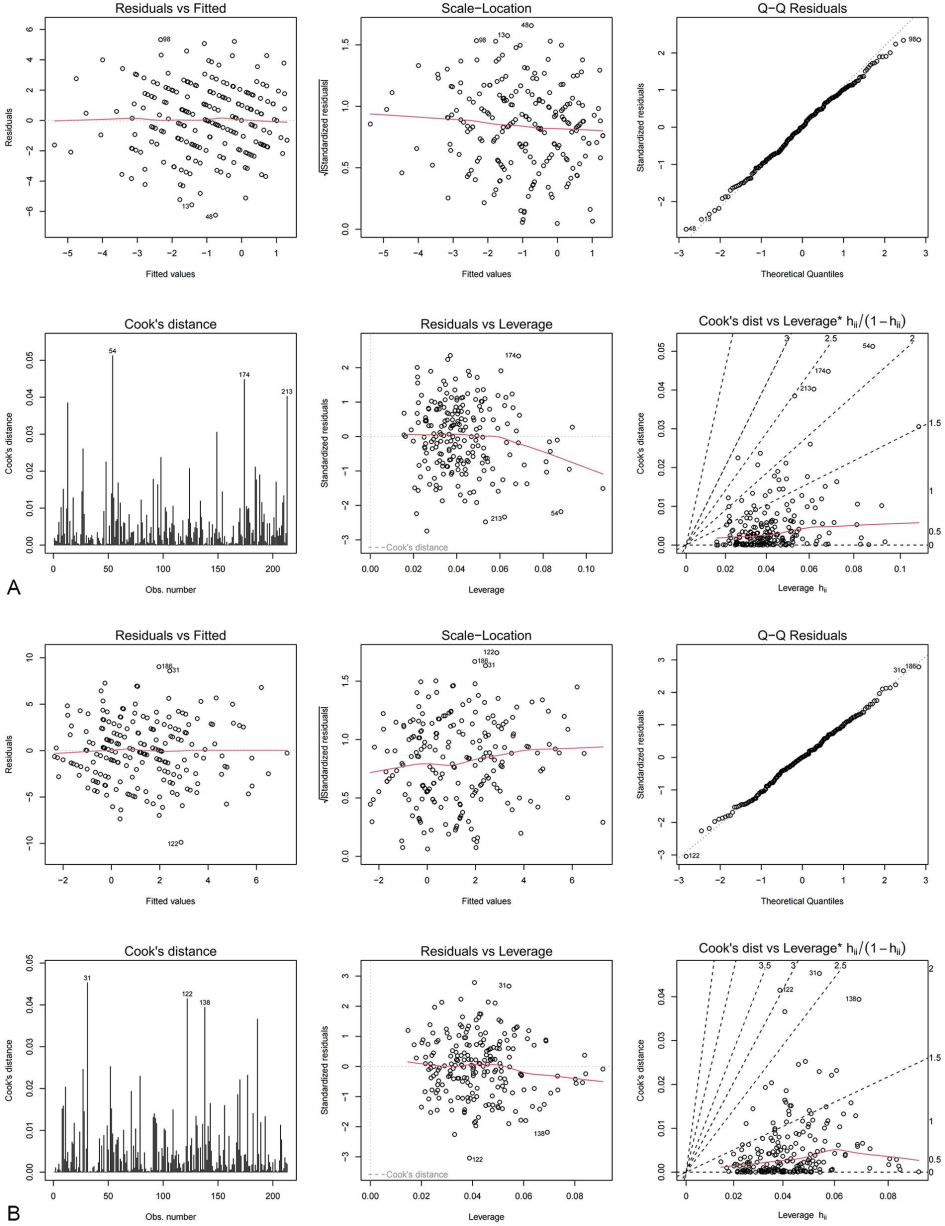
Diagnostic plot of short video viewing duration and *Δ*JOA/*Δ*MCQ-HA multivariate linear regression model. **(a)** *Δ*JOA Model 2 Diagnostic Chart. **(b)** *Δ*MCQ-HA Model 2 Diagnostic Chart.

Multiple linear regression analysis was employed to assess the impacts of short video content exposure and viewing duration on *Δ*JOA among LDH patients. Model 2 diagnostics showed that variance inflation factor (VIF) values for all independent variables ranged from 1.016 to 1.076, indicating no substantial multicollinearity. Residuals followed a normal distribution based on the Shapiro–Wilk test (W = 0.995, *P* = 0.723). The Durbin-Watson statistic was 2.07 (P = 0.68), supporting the assumption of independent residuals (Figure 3A). For content exposure, the frequency of exposure to awareness-motivation content was significantly negatively associated with *Δ*JOA. Specifically, each 1-unit increase in exposure frequency to this content category was associated with an average decrease of 0.31 points in the *Δ*JOA points(*β* = −0.31, 95% CI: −1.11 to −0.44, *P* < 0.001). No significant associations were observed for the other content categories (science education, experience-sharing, and marketing-referral) (all *P* > 0.05).Regarding viewing duration, compared with patients using the platform for <30 min daily, those using it for 30–60 min daily showed an average decrease of 0.16 points in the *Δ*JOA score (*β* = –0.16, 95% CI: −1.77 to −0.006, *P* = 0.037). Patients using it for >60 min daily showed an average decrease of 0.44 points (*β* = –0.44, 95% CI: −3.16 to −1.56, *P* < 0.001). These results suggest that longer daily viewing time is independently associated with poorer lumbar functional outcomes (Table 2).

**Table 2:** Linear regression analysis of short video content exposure, viewing duration, and *Δ*JOA and *Δ*MCQ-HA.

Independent variable	*Δ* JOA				*Δ* MCQ-HA			
	Model 1		Model 2		Model 1		Model 2	
	β (95%IC)	*P* value	β (95%IC)	*P* value	β (95%IC)	*P* value	β (95%IC)	*P* value
Content exposure frequency								
Science education category	−0.02(−0.28,0.40)	0.737	−0.00(−0.35,0.36)	0.965	0.01(−0.43,0.53)	0.845	−0.02(−0.59,0.40)	0.705
Experience sharing category	−0.01(−0.31,0.38)	0.835	0.03(−0.26,0.45)	0.611	−0.11(−0.94,0.03)	0.065	−0.08(−0.82,0.17)	0.197
Alert and mobilization category	−0.34(−1.16,−0.54)	<0.001	−0.31(−1.11,−0.44)	<0.001	0.35 (0.83,1.69)	<0.001	0.35 (0.80,1.74)	<0.001
Marketing lead generation category	−0.04(−0.45,0.22)	0.495	−0.03(−0.42,0.27)	0.652	−0.16(−0.72,0.24)	0.324	−0.07(−0.77,0.20)	0.256
Daily average usage duration grouping								
Group 1 (<30 min)	Reference		Reference		Reference		Reference	
Group 2 (30–60 min)	−0.17(−1.77,−0.11)	0.027	−0.16(−1.77,−0.06)	0.037	0.17 (0.20,2.53)	0.022	0.19 (0.29,2.70)	0.015
Group 3 (>60 min)	−0.42(−3.03,−1.45)	<0.001	−0.44(−3.16,−1.56)	<0.001	0.43 (2.21,4.42)	<0.001	0.44 (2.22,4.47)	<0.001

Model 1: unadjusted.

Mode 2: Adjusted for age, gender, marital status, baseline JOA score, baseline MCQ-HA score, disease duration, educational level, BMI, herniated segment, and symptom type.

A separate multiple linear regression analysis was conducted to evaluate the effects of short video content exposure and viewing duration on *Δ*MCQ-HA scores in LDH patients. Model diagnostics indicated that VIF values ranged from 1.019 to 1.078, suggesting no substantial multicollinearity. Residuals followed a normal distribution based on the Shapiro–Wilk test (W = 0.997, *P* = 0.97). The Durbin-Watson statistic was 1.92 (*P* = 0.27), supporting the assumption of residual independence (Figure 3B). After adjustment for covariates including age, sex, disease duration, and baseline scores, exposure frequency to awareness-motivation content was significantly positively associated with *Δ*MCQ-HA. Specifically, each 1-unit increase in exposure to this content category was associated with an average increase of 0.35 points in the *Δ*MCQ-HA score (*β* = 0.35, 95% CI: 0.80–1.74, *P* < 0.001). In contrast, no significant associations were found for exposure to science education, experience-sharing, or marketing-referral content (all *P* > 0.05). Regarding viewing duration, compared with patients using the platform for <30 min daily, those using it for >60 min daily had, on average, a 0.44-point higher *Δ*MCQ-HA score (*β* = 0.44, 95% CI: 2.22–4.47, *P* < 0.001). This finding indicates that longer daily viewing time is independently associated with a greater increase in health anxiety (Table 2).

**Figure 4:**
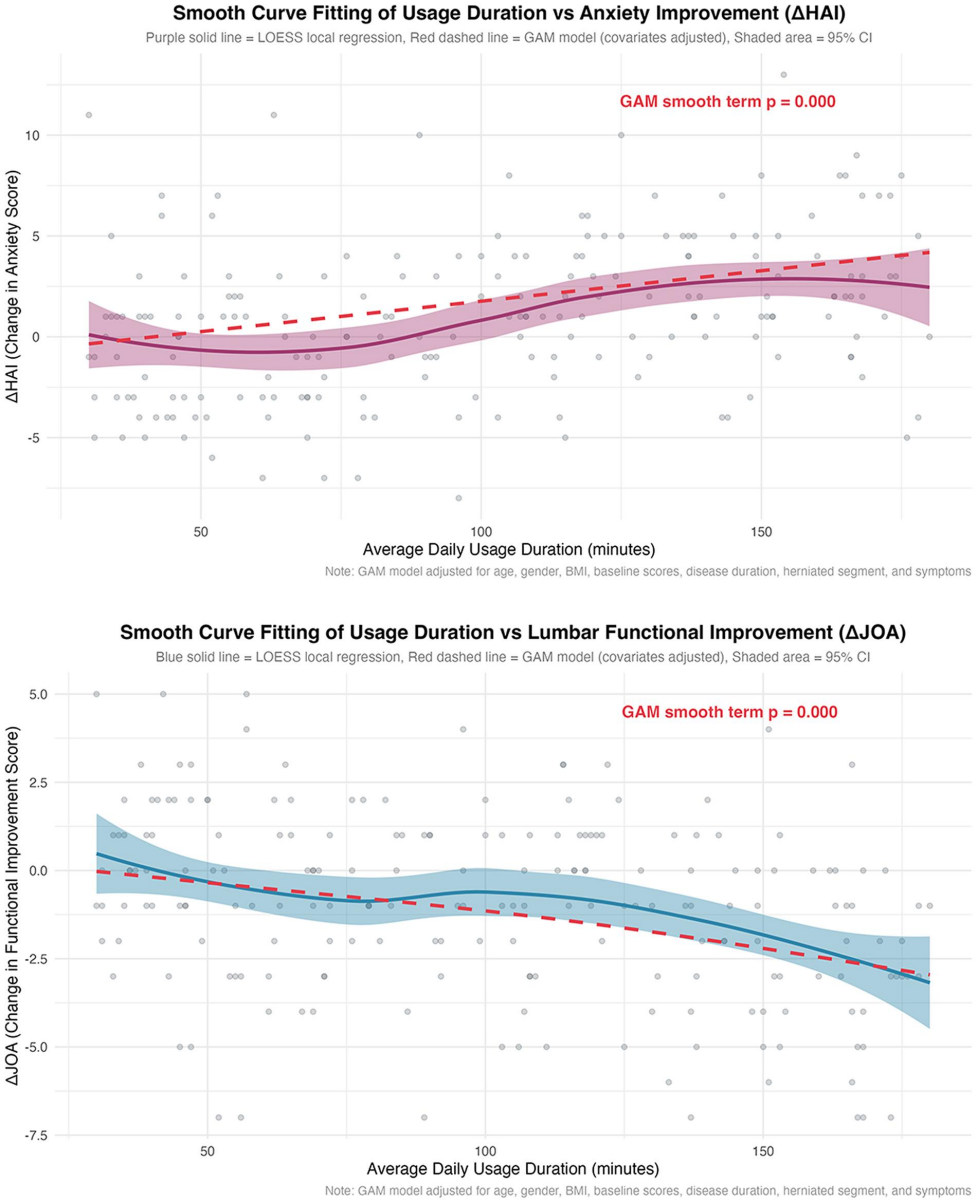
Smooth curve fitting between short video viewing duration and *Δ*JOA/*Δ*MCQ-HA.

Building on Model 2, smoothed fitting curves were utilized to verify the nonlinear relationships between daily viewing duration and *Δ*JOA/*Δ*MCQ-HA. Results revealed a nonlinear negative association between daily viewing duration and *Δ*JOA, as well as a nonlinear positive association between daily viewing duration and *Δ*MCQ-HA (Figure 4).

Given the significant negative association between daily viewing duration and *Δ*JOA confirmed by multiple linear regression, further analysis based on clinical efficacy criteria demonstrated significant differences in rehabilitation outcomes among different viewing duration groups (Table 3). There was a statistically significant difference in the overall distribution of therapeutic effects across the three groups [*χ*^2^ = 18.75, degrees of freedom (df) = 6, *P* = 0.004]. *post-hoc* pairwise comparisons indicated that the effective rate in the >60 min group was significantly lower than that in the <30 min group (adjusted *P* < 0.001).

**Table 3:** Distribution of JOA efficacy grades across different short video viewing duration groups.

Daily viewing time	JOA efficacy grades				*χ*^2^ value	*P* value
	Cured	Marked improvement	Improvement	No improvement		
Viewing Duration Grouping					18.75	0.004
<30 min	4 (7.01%)	10 (18.52%)	19 (33.33%)	24 (42.11%)		
30–60 min	2 (2.63%)	5 (6.58%)	29 (38.16%)	40 (52.63%)		
>60 min	2 (2.5%)	3 (3.75%)	18 (22.5%)	57 (71.25%)		

### 3.5 Subgroup analysis

Subgroup analyses of the current study demonstrated that increased daily average short video viewing duration was significantly associated with reduced lumbar function improvement (*Δ*JOA) and heightened health anxiety (*Δ*MCQ-HA) among patients with Lumbar Disc Herniation (LDH). This association exhibited no significant variations across subgroups stratified by gender, disease duration, educational level, and herniated segment (all interaction *P*-values > 0.05). Notably, the impact of short video usage on lumbar function was more pronounced in patients aged <45 years (*β* = −0.024, *P* = 0.012), whereas health anxiety was more notably affected in those aged ≥45 years (*β* = 0.03, *P* = 0.003), Table 4.

**Table 4:** Subgroup analysis and interaction analysis of the associations between daily average short video viewing duration and *Δ*JOA/*Δ*MCQ-HA.

Variable	*Δ*JOA			*Δ*MCQ-HA		
	β (95%CI)	*P*	*P* For Interaction	β (95%CI)	*P*	P For Interaction
Age			**0**.**359**			**0**.**177**
≥45	−0.024(−0.042,−0.005)	0.012		0.053 (0.027, 0.079)	<0.001	
<45	−0.034(−0.048,−0.021)	<0.001		0.03 (0.010, 0.050)	0.003	
Gender			**0**.**941**			**0**.**586**
Male	−0.031(−0.047, −0.015)	<0.001		0.036 (0.015, 0.058)	0.001	
Female	−0.030(−0.045, −0.015)	<0.001		0.04 (0.016, 0.063)	0.001	
Course of the disease			**0**.**894**			**0**.**456**
≥6 months	−0.032(−0.047, −0.017)	<0.001		0.044 (0.023, 0.065)	<0.001	
<6 months	−0.031(−0.046, −0.016)	<0.001		0.032 (0.008, 0.055)	0.01	
Educational level			**0**.**749**			**0**.**765**
Junior High School or Below	−0.03(−0.050, −0.009)	0.006		0.028(−0.005, 0.061)	0.093	
Senior High School/Technical Secondary School	−0.038(−0.058, −0.018)	<0.001		0.042 (0.014, 0.069)	0.004	
College diploma	−0.022(−0.047, −0.004)	0.093		0.05 (0.018,0.082)	0.003	
Bachelor's Degree or Above	−0.031(−0.054, −0.008)	0.01		0.032(−0.006, 0.069)	0.101	
Herniated Segment			**0**.**637**			**0**.**888**
L4/L5	−0.032(−0.049,−0.015)	<0.001		0.035 (0.011,0.059)	0.005	
L5/S1	−0.026(−0.042,−0.010)	0.001		0.037 (0.012,0.062)	0.004	
Other Segments	−0.042(−0.076,−0.008)	0.017		0.046 (0.004,0.089)	0.034	

The bold represent the P-values follows statistical testing.

### 3.6 Mediation analysis

**Figure 5:**
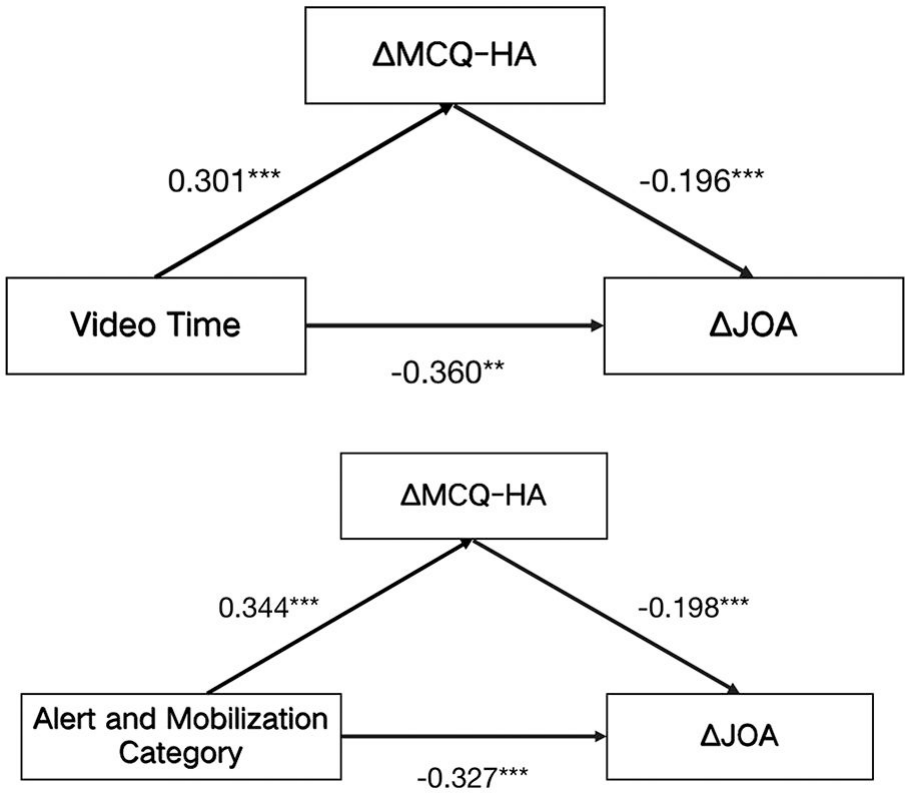
Path diagram of the mediating effect of changes in *Δ*MCQ-HA between short video usage behaviors and *Δ*JOA. *** indicates P < 0.001; **indicates *P* < 0.005.

Mediation analysis was conducted to examine whether changes in health anxiety (*Δ*MCQ-HA) exerted a significant partial mediating role in the relationship between short video usage behaviors and alterations in lumbar function (*Δ*JOA). The total effect of daily short video viewing duration on *Δ*JOA was *β* = −0.031 [95% confidence interval (95% CI): −0.042 to −0.020], with 16.13% of this total effect attributed to the indirect effect mediated by *Δ*MCQ-HA (*β* = −0.005, 95% CI: −0.010 to −0.001). In contrast, the total effect of exposure to warning-mobilization content on *Δ*JOA was *β* = −0.827 (95% CI: −1.152 to −0.503), wherein 20.80% was mediated by *Δ*MCQ-HA (indirect effect: *β* = −0.172, 95% CI: −0.320 to −0.054), as presented in Table 5; Figure 5.

**Table 5:** Mediating effect of changes in health anxiety (*Δ*MCQ-HA) on the association between short video usage behaviors and changes in lumbar function (*Δ*JOA).

Mediating effect	Short video viewing duration [β (95% CI)]	Warning and mobilization content [β (95% CI)]
Total effect	−0.031 (−0.042, −0.020)	−0.827 (−1.152, −0.503)
Direct effect	−0.026 (−0.037, −0.015)	−0.655 (−0.995, −0.316)
Indirect effect	−0.005 (−0.010, −0.001)	−0.172 (−0.320, −0.054)
Mediated proportion (%)	16.13	20.80

## 4 Discussion

This study conducted a 6-month prospective cohort study to systematically investigate the differential impacts of viewing duration and content of lumbar disease-related videos—passively recommended by short video platform algorithms without active user search—on health anxiety and clinical symptoms in patients with lumbar disc herniation (LDH). It also verified the mediating role of anxiety in these associations. The results showed that longer daily average viewing duration of LDH-related content in short videos was associated with increased health anxiety and poor lumbar function recovery after 6 months. Among algorithm-recommended content types, exposure frequency to warning and mobilization-related content had the most significant negative impact on psychological and clinical outcomes, while science popularization and education content, as well as experience sharing content, showed no significant associations. Changes in health anxiety (*Δ*MCQ-HA) played an important mediating role between short video usage behaviors (including viewing duration and exposure to non-standardized content) and changes in lumbar function (*Δ*JOA). These findings provide crucial longitudinal empirical evidence for understanding how the digital information environment, particularly algorithm-driven passive information flow, subtly shapes the psychological state and physiological rehabilitation process of patients with chronic diseases.

In the digital health era, attention resource allocation and threat information processing occur interactively. Influenced by short video algorithm recommendations, individuals may experience increased pressure and inhibition related to their health management behaviors (15). Algorithms continually learn user preferences from behavioral data (e.g., viewing duration, likes, comments) to generate personalized content feeds. This can lead to passive and sustained exposure to high-intensity information pertaining to one's own health status. Such an exposure paradigm may not only reshape health cognitions but, more critically, may alter the psychological underpinnings of health management behaviors via emotional arousal and increased cognitive load. This process can consequently heighten the perception of health-related stress and impair self-regulatory capacity (16).Previous research has demonstrated a significant positive association between the frequency of exposure to illness-related information on digital platforms and levels of pain catastrophizing and health anxiety among individuals with chronic pain (17). This finding offers a key theoretical foundation for understanding the link between short video information exposure and health outcomes in patients with lumbar spinal disorders. This study found a significant positive correlation between changes in health anxiety and daily viewing time (*P* < 0.001). From a cognitive psychology perspective, dedicating substantial daily time to viewing illness-related content—even passively—can result in the continuous allocation of limited working memory resources to illness-related themes. This cognitive preoccupation likely impairs the processing of recovery-relevant information (e.g., proper exercise techniques, positive prognosis cases) and concurrently amplifies attentional bias toward illness-threat cues (e.g., complications, recovery setbacks) (18). More importantly, the reinforcement learning nature of recommendation algorithms can exacerbate this effect. Even brief engagement with specific illness-related content may prompt the platform to persistently deliver similar material, potentially creating a ‘health-threat information silo’ (19). From a health anxiety standpoint, such sustained exposure to negative information may amplify anxiety primarily via two mechanisms. First, a high volume of fragmented threat-related content can disrupt the equilibrium between perceived threat and perceived coping ability, leading to an overestimation of health risks—for instance, misperceiving rare complications as high-probability events (20). Second, the passive consumption of content without professional context (e.g., anecdotal experiences shared by non-medical individuals or exaggerated symptom descriptions) can heighten cognitive uncertainty, which in turn may activate catastrophic thinking (18).

We also observed a significant negative correlation between daily viewing time and lumbar functional outcomes (*P* < 0.001).This association not only reflects a displacement effect, where viewing time supplants time for rehabilitative activities, but also suggests potential influences from dual mechanisms induced by algorithmic recommendations: information overload and cognitive narrowing (21). Specifically, prolonged exposure to algorithmically curated illness-related videos may not only displace time for active rehabilitation but also deplete finite cognitive and emotional resources. This can result in attentional fixation on health-threat information, thereby impairing both the processing efficiency of and the motivation to act upon positive recovery-oriented messages (e.g., rehabilitative guidance) (22). This mechanism aligns with insights from digital health communication research. For instance, a systematic review noted that among social media interventions designed to promote health behaviours, only a minority successfully improved adherence to behaviours such as vaccination (23). This suggests that within complex algorithmic information flows, merely increasing exposure to health information may not necessarily lead to positive behavioral change. If content fails to effectively penetrate users’ cognitive load and trigger deep processing, its impact will be extremely limited.Further mediation analysis indicated that health anxiety partially mediated this relationship. Specifically, daily viewing time was found to exert a negative effect on lumbar functional recovery partly by elevating levels of health anxiety.This finding delineates a clear pathway through which digital engagement can lead to psychological stress that, in turn, impedes physiological function (24). It suggests that algorithmically recommended content may act not merely as passively consumed information but as a persistent psychosocial stressor. This stressor, by amplifying anxiety-driven processes such as catastrophic interpretation of symptoms and excessive worry about recovery, may heighten pain perception sensitivity, foster avoidance of rehabilitative activities, and disrupt normal physiological repair mechanisms. These sequelae could ultimately manifest as impeded recovery of lumbar function. These results provide crucial empirical evidence for understanding the psychophysiological mechanisms by which digital technology engagement can influence physical health outcomes.

Notably, the absence of significant effects for science education and experience-sharing content in this study aligns with the core tenets of self-verification theory. Briñol et al. (25) posit that the influence of information on individual behaviour depends on the dual activation of cognitive and affective validation. While science-based content offers cognitive accuracy, it often lacks the emotional salience and self-relevance characteristic of awareness-motivation content, thereby failing to trigger deep cognitive processing or emotional resonance in patients. Conversely, the high heterogeneity of experience-sharing narratives, combined with the stochastic nature of algorithmic recommendations, may result in content that is poorly matched to an individual patient's specific situation, thus diminishing its potential for self-validation. This finding implies that the health impact of algorithmic information environments is not determined merely by the volume of information but by the content's capacity to activate dual pathways of cognitive and affective validation within the user. This insight offers a theoretical anchor point for future optimizations of algorithmically curated health content. Importantly, our analysis confirmed a significant association between exposure to awareness-motivation content and poorer lumbar functional outcomes (*P* < 0.001). This finding reveals a health risk amplification effect stemming from a ‘negativity bias’ inherent in algorithmic curation. This content category predominantly features negative narratives, such as severe complications, surgical risks, and cases of failed recovery from lumbar spinal disorders. Its dissemination logic is highly congruent with the platform economics of short-video platforms, which prioritize ‘attention capture maximization’ (26). From a neuropsychological standpoint, such content can rapidly induce coupling between the default mode network (DMN) and the ventral tegmental area (VTA). This coupling reinforces viewing behaviour via dopaminergic reward pathways while simultaneously impairing cognitive control functions, thereby exacerbating catastrophic illness-related cognitions (27).

More critically, excessive exposure to awareness-motivation content can reconfigure a patient's illness identity. Prolonged immersion in such narratives may facilitate a shift from viewing oneself as ‘a person in recovery’ to identifying as ‘a potential severe case’. This identity reconfiguration can influence rehabilitation behaviours via a self-fulfilling prophecy mechanism. For instance, excessive worry may lead to avoidance of necessary functional exercises, while unrealistic recovery expectations may breed frustration. Collectively, these responses can initiate a vicious cycle: elevated anxiety fosters behavioural withdrawal from rehabilitation, which impedes functional recovery, in turn further heightening anxiety (28). This mechanism elucidates the mediating role of health anxiety in the link between short-video engagement and lumbar functional changes. Health anxiety thus acts not merely as a psychological affective state but as a critical ‘pathopsychological bridge’ linking digital information exposure to physiological recovery. At its core, health anxiety in this context represents a psychological stress response triggered by algorithmically amplified negative information exposure. This stress response, mediated through activation of the hypothalamic–pituitary–adrenal (HPA) axis, can subsequently modulate pain perception and interfere with tissue repair processes (13). In contrast, the observed ‘ineffectiveness’ of science education and experience-sharing content is not coincidental. Science-based content often suffers from a lack of personalization, failing to address the specific rehabilitation dilemmas of individual patients, which leads to inadequate cognitive validation. While experience-sharing content holds potential for emotional resonance, algorithmic recommendations’ “homogeneous lock-in” may expose patients to narrow rehabilitation experiences, preventing the development of comprehensive health awareness. This phenomenon highlights the “pseudo-personalization” dilemma inherent in algorithmic recommendations (29). Algorithms push homogeneous warning-based content based on traffic preferences, seemingly catering to user needs. In reality, this limits patients’ access to diverse health information, trapping those with lower education levels in negative information echo chambers and exacerbating withdrawal from recovery behaviors (30).

This study has several limitations. First, the relatively single sample source may limit the generalizability of the results. Second, daily viewing duration of LDH and spine-related disease videos relied on self-reported retrospective assessment by participants, lacking objective recording data. Memory bias and time perception deviation may lead to overestimation or underestimation of duration data, making it difficult to reflect true exposure levels. Third, the differential impacts of different recommendation algorithms were not distinguished, hindering the accurate identification of associations between algorithmic technical characteristics and health risks. Fourth, potential moderating variables such as patients’ social support and baseline psychological traits were not included to analyze potential interaction effects. Future research can be expanded in three directions: 1. adopting a multi-center, large-sample cohort design that includes LDH patients of different age groups and disease durations to improve the generalizability of results; 2. integrating algorithm log data to analyze the impacts of different recommendation mechanisms on information exposure quality and health outcomes, providing more precise technical basis for algorithm optimization; 3. conducting interventional studies to verify the effectiveness of an intervention model combining algorithmic content optimization and digital health literacy education, exploring practical pathways to mitigate algorithm-induced health risks.

## 5 Conclusion

This 6-month prospective cohort study confirmed that longer daily viewing duration of disease-related short videos and more frequent exposure to algorithm-recommended warning and mobilization content among LDH patients were associated with poorer lumbar function recovery and higher levels of health anxiety after 6 months. Health anxiety played a partial mediating role in these associations, with a mediated proportion of 16.13% for viewing duration and lumbar function recovery, and 20.80% for exposure to warning and mobilization content and lumbar function recovery. These findings indicate that passive exposure to disease-related content on short video platforms—especially prolonged viewing and high-frequency exposure to warning and mobilization content—significantly hinders the clinical rehabilitation of lumbar function through the key psychological pathway of exacerbating health anxiety. This study not only reveals the specific mechanism by which algorithmic recommendations, as a novel environmental stressor, affect chronic disease rehabilitation but also provides important empirical support for LDH patients’ information self-protection, the optimization of the health content ecosystem on short video platforms, and the consideration of patients’ digital media exposure in clinical practice.

## Data Availability

The original contributions presented in the study are included in the article/Supplementary Material, further inquiries can be directed to the corresponding authors.
